# Identification of psychological constructs for a positive psychology intervention to assist with the adjustment to closed loop technology among adolescents living with type 1 diabetes

**DOI:** 10.3389/fpsyg.2023.1273586

**Published:** 2023-10-13

**Authors:** Sylvia Kruger, Elmari Deacon, Esmé van Rensburg, David Segal

**Affiliations:** ^1^Department of Psychosocial Health, North-West University, Potchefstroom, South Africa; ^2^Optentia Research Focus Area, North-West University, Vaal Triangle Campus, Vanderbijlpark, South Africa

**Keywords:** adjustment to closed loop technology, closed loop technology, positive psychology intervention, qualitative document analysis, type 1 diabetes

## Abstract

**Aim:**

Adolescents have been identified as the group who struggle most with successful adjustment to closed loop technology. This study aims to identify the psychological constructs that should form part of a positive psychology intervention to assist with the adjustment to closed loop technology among adolescents living with type 1 diabetes.

**Method:**

Qualitative document analysis was employed to integrate findings from two documents: a published ongoing intervention study and a recent phenomenological study by the authors. Reflexive thematic analysis was used to identify themes from the documents.

**Findings:**

The following themes were identified as important psychological constructs that aid adjustment: the importance of knowledge and education; the process of positive adjustment to closed loop technology; a positive outlook; and building a relationship with diabetes.

**Conclusion:**

Interventions are needed to assist adolescents in their adjustment to closed loop technology. The psychological constructs identified served as a starting point in designing an effective, evidence-based intervention grounded in data and theory. Knowledge and education, responsibility, identity, positive affect, gratitude, support, and trust are psychological constructs that need to be included in an intervention program.

## Introduction and problem statement

Technology is a key role player in the management of diabetes and has the potential to improve not only medical outcomes but also quality of life (Gonder-Frederick et al., 2016). Closed loop technology involves real-time, glucose-responsive insulin administration, where insulin delivery is partially automated by the pump based on the glucose readings from a continuous glucose monitor (Gonder-Frederick et al., 2016). The medical benefits of these technological devices depend largely on the individual’s engagement with and consistent use of this technology (Gonder-Frederick et al., 2016). The adoption of diabetes technology and its successful use are highly dependent on particular psychological factors (Kubiak et al., 2020). Focusing on the psychological considerations of the adoption of diabetes technology and the successful use thereof is necessary to improve self-management in diabetes care (Gonder-Frederick et al., 2016). A person’s psychological state can have an impact on his or her clinical outcomes and health behavior (Huffman et al., 2015). Negative psychological states, such as depression, correlate with poor treatment outcomes in people living with diabetes (Niemcryk et al., 1990; Anderson et al., 2001; Li et al., 2008). Symptoms of depression among people living with type 1 diabetes are linked to an increased risk of inadequate diabetes self-management, usually ascribed to the fact that depression can lead to difficulties with self-care (Johnson et al., 2013; Schmitt et al., 2021). In contrast to negative psychological states, positive psychological states (e.g., well-being and positive affect) can be viewed as playing a beneficial role in treatment outcomes (Huffman et al., 2015) and are associated with lower HbA1c and fewer complications related to diabetes (Van der Does et al., 1996; Moskowitz et al., 2008; Papanas et al., 2010). Huffman et al. (2015) describe a link between positive psychological states and more involvement in health behavior, where more involvement in health behavior can lead to improved glucose control in people living with diabetes.

It is suggested that positive affect can have a positive influence on conditions where behavioral factors play a role, such as diabetes (Pressman and Cohen, 2005). According to Jaser et al. (2020), inducing positive affect in people living with diabetes may allow them to build intellectual, social, and physical resources. In their study, they hypothesized that by inducing positive affect in people living with diabetes, the use of adaptive coping strategies would be enhanced, diabetes distress would be reduced, and glucose control would improve (Jaser et al., 2020). They argued that emphasizing positive emotions and strengths, instead of focusing on problems, was a more effective way of working with adolescents living with type 1 diabetes. Furthermore, they hypothesized that by using positive affect, it was possible to broaden the range of actions and thoughts in people living with diabetes, and as a result, healthier ways of coping with stress would emerge. The end result would then be to decrease emotional distress and improve diabetes self-management (Jaser et al., 2020).

Positive psychology focuses on cultivating strengths, positive emotions, positive behavior and cognition (Sin and Lyubomirsky, 2009). A prominent theoretical model in the context of positive psychology is the PERMA model. Seligman (2011) designed the PERMA model of well-being, where “PERMA” is an acronym for positive emotions, engagement, positive relationships, meaning, and accomplishment. These constructs work together to bring about the construct of flourishing—an optimal state of functioning (Coffey et al., 2014). These constructs of the PERMA model can further be explained as follows: positive emotions include states of joy, happiness, gratitude, and satisfaction (Fredrickson, 2001; Seligman, 2011). Engagement refers to a state of flow, that is, being completely engaged in an activity (Seligman, 2011). Positive relationships include positive meaningful relationships, social support, and kindness towards others (Ryan and Deci, 2000). Meaning is found in the sense that one’s life is important and makes sense (Steger et al., 2009; Seligman, 2011). Accomplishment refers to the feeling of achievement and mastery (Seligman, 2011).

Interventions that address psychological barriers and facilitating conditions of diabetes technology are needed (Kubiak et al., 2020). Although positive psychology research in the context of type 1 diabetes is limited, several studies have focused on positive psychology interventions in the context of health (e.g., Ogedegbe et al., 2012; Peterson et al., 2012; Jaser et al., 2014), where the principles of positive psychology were used. In this regard, gratitude, self-affirmation, and positive affect (Jaser et al., 2020) have been applied to improve adherence in adults with chronic illness in the context of cardiovascular disease (Peterson et al., 2012) and hypertension (Ogedegbe et al., 2012). In a study on the effects of self-affirmation and positive psychology on adherence to medication among adults with cardiopulmonary disease, an induction of positive affect correlated with a significant improvement in stress and health behavior (Charlson et al., 2007).

Hilliard et al. (2016) argue that an emphasis on positive psychology interventions for people living with type 1 diabetes (e.g., Jaser et al., 2014) may be beneficial to them, as it would focus on their strengths and their families’ strengths to promote resilience. Constructs of well-being, such as positive affect and optimism, have been associated with improved medical outcomes for individuals living with diabetes (Massey et al., 2018). A positive state can improve a person’s psychological, social, and physical resources (Aspinwall and Tedeschi, 2010; Yi-Frazier et al., 2012), and positive affect is associated with improved problem-solving skills and coping (Aspinwall and Tedeschi, 2010). Positive psychological states are associated with increased motivation, goal-directed behavior and self-control and have the potential to influence health behavior (Aspinwall, 1998; Fishbach and Labroo, 2007).

Limited research has been conducted on interventions for adolescents living with type 1 diabetes. According to the National Institute of Health and Care Excellence (2015), adolescence can be a period of non-adherence to diabetes self-management behavior and, as a result, worsening glucose control. Adolescence is viewed as a critical time for individuals living with type 1 diabetes, because they must navigate normal developmental challenges in addition to the complexities of diabetes (Skinner et al., 2000). The patterns of diabetes management established during adolescence typically continue into adulthood (Bryden et al., 2001). Therefore, it is necessary to develop a self-management care plan that is compatible with each individual (Smith et al., 2018).

Diabetes self-management has increasingly become more dependent on technology (Tanenbaum and Commissariat, 2022). It is important to recognize the interaction between positive experiences for the person living with diabetes and technology success, as well as the fact that the biggest barrier to using technology to its full potential is human factors that limit the effective use thereof (Gonder-Frederick et al., 2016). The use of diabetes technology has not been sufficiently studied in the adolescent population (Faulds et al., 2021). No intervention program focusing on adjustment to closed loop technology could be found in the literature search. Increasing people’s access to interventions can help facilitate the uptake of, effective adjustment to and continued use of diabetes technology (Gonder-Frederick et al., 2016). It is recommended that medical and psychological factors should be considered in diabetes to facilitate better integration of diabetes technology into the everyday lives of people living with diabetes and to benefit from technological advances (Gonder-Frederick et al., 2016).

The unified theory of acceptance and use of technology (UTAUT) is a theoretical model relevant to the use and adoption of diabetes technology (Venkatesh et al., 2003). The UTAUT emphasizes the important role of the individual’s beliefs, attitudes, and perceptions in making decisions about using technology (Venkatesh and Davis, 2000). According to the UTAUT model, effort expectancy (the ease of use of the technology), performance expectancy (in what way does using the technology help the individual attain improved performance), social influence (a person’s perception that important people in their life believe they should use the technology) and facilitating conditions (a person’s perception of how well the organizational and technical infrastructure supports the use of the technology) are the most important factors of behavioral intention (Venkatesh et al., 2003; Zhang et al., 2019).

The aim of the qualitative document analysis was to identify the psychological constructs that should form part of a positive psychology intervention program for adolescents who are adjusting to closed loop technology. Adjustment within the context of the current manuscript, is viewed as psychological adjustment to closed loop technology. Thus, how individuals adapt emotionally and psychologically to the use of closed loop technology, for example, feelings of acceptance, trust in the technology and utilising the technology to the best of its ability in order to optimise diabetes management. Adjustment thus refers to the successful integration of closed loop technology into an adolescent’s life for optimal performance and addressing the psychological and emotional aspects to ensure a holistic approach to diabetes management. This aim was achieved by combining findings from the literature and the experiences of adolescents living with closed loop technology. The research question was: What psychological constructs should form part of an intervention for adolescents living with type 1 diabetes and using closed loop systems?

## Method

Qualitative document analysis was employed in the identification of the psychological constructs. This method refers to a systematic procedure for evaluating documents and identifying themes within the documents (Bowen, 2009). The study was approved by the Health Research Ethics Committee of the North-West University, South Africa (NWU-00266-21-A1).

### Data

A published ongoing intervention study (Jaser et al., 2020) and a phenomenological study by the current authors (Kruger et al., under review)^3^ were used as data for the qualitative document analysis. Both studies emphasize the need for interventions for adolescents living with type 1 diabetes. It was decided to utilize the ongoing trial by Jaser et al. (2020) due to a lack of availability of published positive psychology intervention studies for adolescents living with type 1 diabetes. Jaser et al. (2020) build on previous work, namely pilot studies testing posting psychology interventions for adolescents with type 1 diabetes (Jaser et al., 2014; Zhang et al., 2018). Both of these pilot studies indicate the suitability of delivering interventions to adolescents by means of text messages. However, the interventions could not detect the effects of the intervention; hence, work in this area remains to be done. In one pilot study—Jaser et al. (2014)—a significant correlation was found between adolescents’ levels of positive affect and measures of adherence, including self-reporting and glucose monitoring device downloads. The aim of the current study is to identify the psychological constructs that should form part of a positive psychology intervention to assist with the adjustment to closed loop technology among adolescents. To achieve this aim, the authors employed two distinct documents, each containing unique types of data. Document 1 was chosen due to its specific focus on a positive psychology intervention for adolescents. This document helped the authors establish a foundational understanding of an existing positive psychology intervention in the context of type 1 diabetes. On the other hand, the second document focusing on adolescents’ experiences of adjustment to closed loop technology brought a different dimension to our study, as it contained an in-depth description of those directly adjusting to closed loop technology. This second document enriched our analysis by offering qualitative perspectives and real-life examples. The integration of these two documents into the data analysis process was a deliberate choice to foster a more comprehensive exploration of the research question. By considering both types of data, we aimed to mitigate potential biases and limitations associated with relying solely on one data source.

Document 1 is the study by Jaser et al. (2020). For the qualitative document analysis, the authors analysed the published manuscript by Jaser et al. (2020) and did not have access to the full intervention or raw results. Document 1 included background on the positive psychology framework utilised when developing the intervention, the study aims, the research design and methods, the study population, the recruitment process, an outline of the intervention, the outcome measures and the results. The above provided a rich account for the authors to utilise document 1 as a part of the qualitative document analysis. This positive psychology intervention study was an ongoing randomized control trial comparing diabetes education and a positive affect intervention based on text messages in a diabetes education control group with the aim of reducing diabetes distress in adolescents with type 1 diabetes and to improve their diabetes outcomes. The targeted sample size of this intervention was 200 adolescents diagnosed with type 1 diabetes and their caregivers (100 participants for each group). Thr1VE! was developed from a series of pilot studies of positive psychology interventions in the context of adolescents living with type 1 diabetes (Jaser et al., 2014; Zhang et al., 2018). Theoretically, the intervention was grounded in Fredrickson’s (2001) broaden-and-build theory of positive emotions, with the hypothesis that inducing positive affect leads to the broadening of thoughts and actions, thereby increasing healthy responses to stress, decreasing diabetes distress and improving diabetes self-management. The positive psychology intervention THR1VE! was developed for adolescents between the ages of 13 and 17. The intervention included a health behavior contract, where the participants had to commit to a behavior they wanted to change by writing down a goal, which was introduced during an individual session with a research assistant. They were encouraged to share this goal with their family and friends. The caregiver participants also had one session with the research assistant. The intervention also included worksheets on the following topics: gratitude, self-affirmation, writing about positive experiences, accomplishments, and diabetes education material. Automated text messages were also sent to the participants to remind them to reflect on gratitude. Worksheets were also provided to the caregivers, outlining the effect of positive messages on adolescents’ behavior. The goal of the worksheet was for the caregivers to acknowledge the adolescents’ strengths and separate these from diabetes. The intervention was delivered remotely, except for the initial session with the research assistant. Automated text messages were sent to prompt the adolescent participants to reflect on gratitude and their top value for 8 weeks and included the following content: mood booster content, important values, gratitude, and unexpected gifts. The plan was for the intervention study to be delivered to the participants for 12 months.

Document 2 is the study by Kruger et al. (under review, see footnote 1). This interpretative phenomenological analysis (IPA) study aimed to obtain an in-depth understanding of the experiences of adjustment for adolescents living with closed loop technology. Within the study, successful adjustment was viewed as adolescents using closed loop technology who achieved an HbA1c of <7% and a time in range (TIR) of >70%. As IPA aims to understand experiences in depth, a small sample was selected, in line with the literature on IPA (see Smith et al., 2009). A relatively homogenous sample is recommended by the literature on IPA (Smith et al., 2009); therefore, the researchers recruited five participants living with type 1 diabetes between the ages of 15 and 18, who had successfully adjusted to closed loop technology. In line with the literature on IPA (Smith et al., 2009), open-ended interviews lasting approximately 45 min were conducted. An interview schedule with open-ended questions and possible prompting questions was developed in line with the research question and aim. The following questions and requests were posed (excluding prompting questions): (1) please tell me about yourself. (2) Please tell me more about the role of diabetes in your life. (3) Please tell me more about living with closed loop technology. (4) Is there anything else about your journey with diabetes that you would like to share today? After the data collection, the interviews were transcribed, analyzed and interpreted using the steps outlined by Smith et al. (2009). The final results were then written up. Although the authors have access to the raw data and transcripts supporting the final results of document 2, only the written manuscript was used in the qualitative document analysis, in line with document 1. Within document 2, the recruitment process, the participants’ context, the interview process and questions posed to the participants, the methodology, data analysis and results of the study are discussed. With all the themes discussed in document 2, verbatim extracts were used to support all interpretations made, in order for the reader to be able to verify the interpretations made.

The following themes were constructed: learning to trust the technology; making diabetes visible; building a relationship with diabetes; the value of support; and experiencing positive outcomes. The study showed that psychological factors play a crucial role in people’s successful adjustment to closed loop technology and it is essential to focus on these psychological factors to experience the optimal benefits of the technology. Within the identified themes, the following psychological factors were identified: perception, attitude, willingness to learn, and motivation. In the study, the participants emphasized the importance of engagement and management for the person living with diabetes. Adjustment to closed loop technology is facilitated by the processes of learning to trust the technology, having access to visible glucose data, building a relationship with diabetes, support, and experiencing positive outcomes. Adjustment is further facilitated by being able to “see” diabetes, which leads to participants being able to make better diabetes management decisions and trust the technology, leading to improved diabetes management. In turn, improved management and control of diabetes lead to acceptance of diabetes technology and the incorporation of diabetes into one’s identity.

### Data analysis

Within qualitative document analysis, thematic analysis is viewed as an appropriate means of analyzing data (Morgan, 2022). For this qualitative document analysis, reflexive thematic analysis (see Clarke and Braun, 2013; Braun and Clarke, 2020) was conducted with the aim of establishing themes within the data. The following process was followed: first, the authors familiarized themselves with the data by reading and re-reading the two documents individually. As stated above, the authors did not utilise the raw data, but rather the two documents in article format. The documents were read and re-read to become intimately familiar with the content in order to identify patterns and similarities in the data. Second, they generated codes for important features of the data relevant to the research question that guided the analysis, the codes included short phrases that encapsulate important concepts within the data. The entire articles were utilised, however within document 1, the focus was on the content of the intervention, while in document 2, the focus was on the themes identified as crucial to effective adjustment. Third, the authors constructed possible themes by identifying similarities in the codes. This involved searching for recurring concepts in the data. Fourth, the authors reviewed the generated themes with regard to the codes and the transcripts. This involved revisiting the codes and both documents to ensure that the themes accurately reflect the content of both documents. Fifth, they named the final themes and developed a description of each theme, while referring back to both documents analysed. The final step was producing the final report and disseminating the findings via the current manuscript.

The first author analysed the data. The second author was the co-coder and independently verified the results. The co-coder was a means of validating the themes identified and increasing the reliability of the study as well as bringing a different perspective to the themes and identifying any potential blind spots the first author might have missed. The third and fourth author read through the analysis to verify the results. Peer discussions were also held on the results and interpretations made. Throughout the study, a reflective journal was kept by the first author in order to ensure that the data was not biased. In this way, the author was constantly aware of her own assumptions, emotions, views, and personal biases regarding the research topic. Within document 2, verbatim extracts were used to support all interpretations made, to ensure that the results were grounded in the data. With the qualitative document analysis, the authors referred back to both document throughout the analysis to ensure that the themes generated were based on the data.

## Findings of the qualitative document analysis

Based on the analysis of the two documents, four main themes were constructed, as summarized in Table 1. From the themes, psychological constructs that should form part of a positive psychology intervention program were identified and are described in the “Discussion” section. Within the current section, the themes that were constructed from the qualitative document analysis are described.

**Table 1:** Themes identified in the two analyzed documents.

Theme	Sub-themes
Theme 1: the importance of knowledge and diabetes education	
Theme 2: the process of positive adjustment to closed loop technology	Commitment to and responsibility for managing type 1 diabetes well
	Trusting the technology is necessary to integrate the technology into everyday life
Theme 3: positive outlook	Positive affect
	Gratitude
Theme 4: building a relationship with diabetes	Identity
	Support

### Theme 1: the importance of knowledge and diabetes education

Both documents emphasize that it is crucial to receive education about diabetes in order to successfully manage the condition. According to the literature, diabetes management education is essential for people to effectively live with diabetes (Funnell and Anderson, 2004; Ghisi et al., 2022). Successful diabetes management requires sufficient knowledge on the part of the person living with diabetes; this knowledge includes the goals of treatment, the effect diabetes has on the body and the factors that can influence glucose levels (Gonzalez et al., 2016). It is crucial for people who live with diabetes to be treated as partners in their healthcare and diabetes management (Phiri et al., 2022).

In document 1 (Jaser et al., 2020), an education packet is provided that includes basic diabetes constructs, such as glucose levels, exercising, travelling and HbA1c, developed from the American Diabetes Association website. The participants of document 2 (Kruger et al., under review, see footnote 1) emphasized that an important part of adjusting to living with diabetes and closed loop technology is a willingness to learn and “trial and error,” in other words, learning through experience and finding what works for the individual. An important aspect for the participants in document 2 (Kruger et al., under review, see footnote 1) was to integrate what they have learnt in the past with adjusting to the closed loop system. This highlights a process of adjustment that occurs over time. The participants reported that support received from healthcare providers, training on the closed loop system and practical support from caregivers with, for example, helping with infusion sets facilitated effective adjustment. This finding highlights the importance of education in the context of managing diabetes and effectively adjusting to closed loop technology.

Most individuals living with type 1 diabetes are exposed to knowledge about diabetes management during diabetes education provided at their healthcare practitioner’s office. However, it is not known to what extent self-management practices are integrated into their daily life, as most people living with type 1 diabetes do not attain optimal control (Weller et al., 2016). It is recommended that adolescents and their caregivers receive ongoing education about diabetes self-management and diabetes technology (Dowling, 2021). It has been found that closed loop systems can be initiated by virtual training programs and that virtual training programs can improve glycemic control (Petrovski et al., 2021). This therapeutic approach to diabetes education is one of the fundamentals of a successful program. In this approach, the professional and the person living with diabetes become partners in learning how to integrate diabetes into their daily lives (Heller et al., 2020).

### Theme 2: the process of positive adjustment to closed loop technology

In the process of positive adjustment, two sub-themes were constructed, namely (1) commitment to and responsibility for managing type 1 diabetes well and (2) trusting the technology is necessary to integrate the technology into everyday life. In order to positively adjust to closed loop technology, the adolescent participants in document 2 (Kruger et al., under review, see footnote 1) had to commit to particular diabetes management behaviors and take responsibility for their diabetes management. In time, this led to them trusting the closed loop system and integrating it into their everyday life.

#### Commitment to and responsibility for managing type 1 diabetes well

People with type 1 diabetes often face a particularly challenging time during adolescence, as blood glucose levels are higher in this period (Johnson et al., 2013) and their responsibility increases in terms of managing the condition more independently from their caregivers (Ingerski et al., 2010). Several physical and emotional changes take place during the adolescent phase due to puberty (Best and Ban, 2021). During this period, adolescents also seek independence, and usually the roles in diabetes care change—where the caregiver initially took responsibility for the management of diabetes, the adolescent now takes responsibility for managing the condition (Ingerski et al., 2010). Adolescents have been identified as the group that experiences the most difficulty with glycemic control among all people living with type 1 diabetes (Messer, 2019), with only approximately 20% of them meeting their glycemic targets (Miller et al., 2015).

Managing type 1 diabetes well requires constant engagement in self-management behavior (Johnson et al., 2013), and an unwillingness to participate in health behavior is viewed as an obstacle to managing diabetes successfully (Rollnick et al., 2008). This challenge is addressed in document 1 (Jaser et al., 2020) by the use of a health behavior contract where the participants had to commit to a type of behavior they wanted to change by writing down a goal. The participants were then encouraged to share this goal with family and friends. The aim of the health behavior contract was to improve the participants’ adherence to diabetes management behavior and motivate them to manage the condition well. Responsibility was further reinforced by encouraging the participants to share the goal with their family and friends.

Positive psychological states are viewed as facilitating conditions and are associated with increased motivation, goal-directed behavior, and self-control (Aspinwall, 1998; Fishbach and Labroo, 2007). This was evident in document 2 (Kruger et al., under review, see footnote 1) where the participants were of the opinion that they felt optimistic when their diabetes was managed to the best of their ability. The participants of document 2 (Kruger et al., under review, see footnote 1) pointed out that behavioral skills were important for managing the condition, for example counting carbohydrates and administering a bolus dose at the correct time. Within this study, motivation and commitment to managing diabetes well were identified as important psychological factors in adjusting to closed loop technology. Likewise, adopting the belief that it is important to manage diabetes, adopting a self-management routine and consistently applying the self-management routine were identified as important factors in adjusting to this technology. Within the context of any chronic illness, understanding what can and what cannot be controlled is an essential aspect of adapting to living with the illness (Reid, 1984). Previous studies have shown that personal beliefs about diabetes are linked to self-management practices (Hampson et al., 1990; Chesla et al., 2000).

Messer (2019) points out that many adolescents who have access to closed loop technology choose not to use the device consistently. Closed loop technology is a valuable tool for people with diabetes and holds promise to improve their glycemic control. Within document 2 (Kruger et al., under review, see footnote 1), the participants emphasized that although the closed loop system is a helpful management tool, ultimate success in using it depends on how the person living with type 1 diabetes engages with the device, as well as their self-management behavior. Hence, the responsibility of managing diabetes lies with the person living with diabetes. It was evident from all the participants’ accounts that conscientiousness played a significant role in their ability to adjust to closed loop technology, as they were all committed to managing their diabetes well and consistently applied the diabetes management routine. From document 1 (Jaser et al., 2020) and document 2 (Kruger et al., under review, see footnote 1), it is concluded that negotiating responsibility for diabetes self-management is a necessary part of adjusting to closed loop technology.

#### Trusting the technology is necessary to integrate the technology into everyday life

As document 1 (Jaser et al., 2020) focused on type 1 diabetes in general, and no other positive psychology intervention study could be found on closed loop technology, closed loop-specific content from document 2 (Kruger et al., under review, see footnote 1) is also included in this discussion. Within the study reported on in document 2 (Kruger et al., under review, see footnote 1), all the participants initially struggled to trust the closed loop system. It was essential for the participants to obtain trust in the closed loop system in order to integrate the technology into their daily lives, and they had to adopt the belief that the closed loop system would be beneficial for them. Glucose numbers are seen by some adolescents as a signal of brokenness or disability, rather than an expression of self-awareness, and this can have an impact on their self-esteem, identity, and self-confidence (Kubiak et al., 2020). Many adolescents report feeling guilty and like a failure when they have high glucose level readings (Palladino and Helgeson, 2012). Furthermore, at times, they avoid performing diabetes care in the presence of their peers (Palladino and Helgeson, 2012). All the participants of document 2 (Kruger et al., under review, see footnote 1) made reference to the fact that although continuously having access to glucose data was beneficial, it could also lead to feelings of information overload. Trust has been identified as a crucial part of the process of adjustment, and for the adolescents reported on in document 2 (Kruger et al., under review, see footnote 1), having access to visible glucose data increased their trust in the closed loop system. This finding suggests that if an individual who uses closed loop technology does not have trust in the technology, their ability to successfully adjust to using the technology will be hindered.

### Theme 3: positive outlook

The sub-themes of theme 3 were (1) positive affect and (2) gratitude. Constructs of psychological well-being, such as self-efficacy, gratitude, and positive affect, have been linked to better health outcomes for people with chronic conditions (Massey et al., 2018). A positive outlook for the adolescents living with type 1 diabetes in document 2 (Kruger et al., under review, see footnote 1) was evident in their experiences of positive affect and gratitude, which contribute to good diabetes outcomes relevant to promoting well-being, despite risk factors (Steinberg et al., 2017).

#### Positive affect

Constructs of well-being, such as positive affect and optimism, have been found to correlate with better medical outcomes for people living with diabetes (Massey et al., 2018). In document 1 (Jaser et al., 2020), the authors hypothesize that by enhancing positive affect in people with diabetes, adaptive coping strategies would be enhanced, diabetes distress would be reduced and glucose control would improve. They argue that emphasizing positive emotions and strengths, instead of focusing on problems, is a more effective way of working with adolescents living with type 1 diabetes. This does not imply suppressing emotions, but rather strengthening positive affect. According to Fredrickson’s (2001) broaden-and-build theory, positive affect can increase one’s skills to utilize complex and creative coping strategies by enlarging one’s skills in cognition and attention. In contrast, experiences that cause negative affect can often decrease the number of responses available, such as fear that leads to avoidance (Fredrickson, 2001). The authors of document 1 (Jaser et al., 2020) hypothesized that by inducing positive affect, it was possible to broaden the range of actions and thoughts among people living with diabetes, and as a result, healthier ways of coping with stress would emerge. The end result would then be to decrease emotional distress and improve diabetes self-management.

The participants of document 2 (Kruger et al., under review, see footnote 1) displayed an optimistic attitude and a sense of personal growth in their experience of adjusting to closed loop technology, despite being aware of the challenges that they experienced. Two psychological factors were reported as a result of successful adjustment, namely improved quality of life and positive growth. All the participants praised the closed loop system for improved quality of life, for example giving a warning when blood glucose is out of range. Moreover, they stated that the technology provided them with the opportunity to live a more normal life.

In document 2 (Kruger et al., under review, see footnote 1), some participants expressed a need to assist others through their own experience of living with diabetes. The participants also reported that living with diabetes made them stronger, increased their maturity and gave them a sense of responsibility.

#### Gratitude

As a part of the intervention in document 1 (Jaser et al., 2020), the participants were asked to identify gratitude on a worksheet and also identify, from a list, three items they viewed as important. The theme of gratitude is highlighted in document 2 (Kruger et al., under review, see footnote 1), where the participants expressed gratitude for closed loop technology and stated that they viewed it as freeing in comparison to multiple daily injections. They described gratitude for the closed loop system, as they were of the opinion that it helps them thrive in life. In document 2 (Kruger et al., under review, see footnote 1), gratitude is viewed as an outcome of successful adjustment. Schache et al. (2019) implemented a gratitude journaling intervention for adolescents with type 1 diabetes. They indicated that it was a cost-effective psychosocial intervention that had the potential to improve physical and psychological health outcomes. Diabetes self-management has increasingly become more dependent on technology. It is important to recognize the interaction between positive experiences for the person living with diabetes and success in using diabetes technology, as well as the fact that the biggest barrier to using technology to its full potential is human factors that limit the effective use thereof (Gonder-Frederick et al., 2016).

### Theme 4: building a relationship with diabetes

In building a relationship with diabetes, two sub-themes were constructed, namely (1) identity and (2) support.

#### Identity

The developmental tasks of adolescents include the development of identity and autonomy, becoming socially responsible and acquiring a set of values to guide their behavior (Foster, 2009; Montali et al., 2022). During this developmental stage, independence from caregivers is developed and various biological and hormonal changes take place (Jaser, 2010; Carlsund and Söderberg, 2019). Erikson (1968) argues that adolescents’ primary task is to develop their identity. Interaction with peers contributes to the development of identity (Foster, 2009).

Montali et al. (2022) have found that type 1 diabetes can challenge an individual’s self-concept and personal identity. In an education program, people living with diabetes should be able to learn to fit diabetes into their everyday lives (Heller et al., 2020).

In document 1 (Jaser et al., 2020), each of the participating adolescents was requested to write down their best personal value and the reason why this value is important to them. They were then asked to write about any positive experience or accomplishment, and the value that they selected was used for self-affirmation. This exercise touched on the concept of identity, where adolescents can identify a positive part of themselves. In document 2 (Kruger et al., under review, see footnote 1), it is emphasized that diabetes is a part of the person’s identity, but they are not defined by diabetes. For the participants in document 2 (Kruger et al., under review, see footnote 1), taking responsibility for diabetes management led to acceptance of diabetes, without having a negative influence on their identity. Another part of incorporating diabetes into their identity was the realization that diabetes could not be managed perfectly. The participants made reference to the acceptance of diabetes as a part of who they are and the importance of deciding to manage and accept the condition.

#### Support

Social support in the context of diabetes self-management includes the fulfilment of two needs of the person living with type 1 diabetes, namely practical aspects of managing the condition and emotional support (Montali et al., 2022). Mattacola (2020) has found that too much practical advice is not always viewed as supportive and some adolescents value non-diabetes-specific support, such as support from their caregivers or their peers.

Support was highlighted in both documents. Document 1 (Jaser et al., 2020) emphasized caregiver involvement by having one session with caregivers and the research assistant, where a caregiver praise worksheet was completed with the aim of enhancing positive affect and increasing positive interaction between the adolescent and the caregiver. The goal was to recognize the adolescents’ strengths as separate from diabetes. The caregivers received weekly reminders in the form of text messages to give positive messages to the adolescents living with diabetes.

Within document 2 (Kruger et al., under review, see footnote 1), support was identified on two levels: practical and emotional. On a practical level, the participants highlighted the support received from healthcare professionals, adequate training and practical support from their caregivers, for example assisting with self-management tasks. On an emotional level, support, such as time spent with friends and family and knowing that one has a support structure, was identified as critical to their successful adjustment to closed loop technology. The participants also stated that social media, especially in the form of role models of other people living with diabetes, was useful in their journey towards successful adjustment to closed loop technology. Some participants also expressed a need to give back or assist others who are living with type 1 diabetes. The theme of support in document 1 (Jaser et al., 2020) and document 2 (Kruger et al., under review, see footnote 1) suggests that adolescents’ effective adjustment to closed loop technology is facilitated by support from significant others and healthcare providers.

## Discussion of the psychological constructs that should form part of a positive psychology intervention

Within the four themes and their sub-themes identified in the document analysis, five psychological constructs that should be included in a positive psychology intervention focusing on effective adjustment to closed loop technology were identified. The five constructs are in line with the UTAUT and Seligman’s PERMA model and overlap with these theoretical models. The constructs are knowledge, responsibility, identity, positive affect, gratitude, support, and trust. The psychological constructs as discussed below are not an intervention and the current authors will develop an intervention utilising the below constructs as the foundation.

The UTAUT is important in the context of the acceptance of closed loop technology. As identified in document 2 (Kruger et al., under review, see footnote 1), similar to the construct of effort expectancy, the participants had to adopt the belief that the technology was easy to use and led to better diabetes management. All the participants of document 2 (Kruger et al., under review, see footnote 1) viewed the technology as beneficial for their health and indicated that having access to visible glucose data enabled them to improve their diabetes management, in line with the construct performance expectancy. Similar to the social influence construct, the participants found that emotional and practical support facilitated their adjustment to closed loop technology. The facilitating conditions identified include having a continuous visual representation of one’s glucose data, taking personal responsibility, positively integrating diabetes and closed loop technology into one’s identity, and having a positive attitude. Seligman (2011) developed the PERMA model of well-being, which consists five elements of well-being (positive emotion, engagement, relationships, meaning, and accomplishment). The elements of the PERMA model are evident in the psychological constructs identified below.

### Construct 1: knowledge and education

As identified in the first theme, “the importance of knowledge and education,” having sufficient knowledge about how to manage diabetes is crucial. It is recommended that an intervention program includes an educational component on basic diabetes constructs, for example the importance of good glucose control, and knowledge of diabetes self-care behavior. As identified through the document analysis, trust was facilitated by having access to visible glucose data; it is thus recommended that knowledge about the construct of time in range is included in an intervention. As identified through the qualitative document analysis, having access to visible glucose data is beneficial, but sometimes it can also be overwhelming. It is recommended that psychological topics, such as diabetes burnout (Abdoli et al., 2021), and additional resources that adolescents and their caregivers can access at their own time are included in an intervention. Within the knowledge construct, the PERMA construct of accomplishment is addressed, as well as the UTAUT construct of performance expectancy. With more knowledge, adolescents, and their caregivers will feel empowered and accomplished, have more trust in the closed loop system and view the system as beneficial for their health, which will facilitate adjustment. Examples of the educational content that should be provided include: information on the PERMA constructs and how this can be applied in daily life, information on time in range, benefits of good glucose control, nutritional information and diabetes burnout.

### Construct 2: responsibility and identity

Within the second and fourth themes, “the process of positive adjustment to closed loop technology” and “building a relationship with diabetes,” the psychological constructs of responsibility and identity were identified. During adolescence, adolescents seek autonomy and independence (Lam et al., 2014). Chiang et al. (2014) emphasize that important tasks for adolescents living with type 1 diabetes are the development of independence and identity; therefore, healthcare providers and family members should aim to support adolescents’ transition to independence and increased responsibility. Identity development occurs continuously throughout a person’s lifetime (Timler et al., 2019). The physical and psychological aspects of living with diabetes should be taken into account when identity development among adolescents is considered (Adal et al., 2015). A clear sense of identity can help people living with type 1 diabetes cope with their diabetes appropriately and may protect them from psychological problems (Verschueren et al., 2019). It is recommended that, in an intervention for adolescents living with type 1 diabetes, the construct of responsibility is addressed by, for example, including information and activities on setting personal goals or activities such as envisioning their best version of themselves. Identity development is crucial during the adolescent phase, and there should be a focus on integrating type 1 diabetes into their identity in interventions for adolescents living with type 1 diabetes. By setting and achieving goals and by integrating diabetes and the closed loop system into one’s identity, the following constructs from the PERMA model and the UTAUT are touched on: accomplishment, engagement, and facilitating conditions.

By taking responsibility for managing type 1 diabetes, it is possible for adolescents to engage in better self-management behavior, which will increase their diabetes health behavior and result in a feeling of accomplishment. When adolescents accept more responsibility for managing their diabetes, it is possible that they will experience being empowered and fully engaged in the management of the condition. Within the document analysis, it was found that it is essential to integrate diabetes and diabetes technology into adolescents’ view of themselves, and this can be viewed as an essential facilitating condition in their adjustment to closed loop technology. This is mirrored in research by Raymaekers et al. (2019), who describe that integrating diabetes into one’s identity plays an important role in coping with diabetes. Within the context of an intervention, an example of enhancing responsibility could be to provide education on goal setting and by tasking adolescents with writing down one behavior that they would want to change within the context of diabetes and keep track of their progress on a cell phone or by writing it down on a daily basis. It could be encouraged to record any progress/ successes and to share their goal with a caregiver. Caregivers could be tasked with praising any positive change or small accomplishment that their adolescent had. Adolescents could also be asked to envision their best possible self when achieving their health goals as well as identifying the step to support their goals.

### Construct 3: positive affect and gratitude

The importance of positive affect and gratitude was evident in theme 3, “positive outlook,” of the qualitative document analysis. These constructs should be included in an intervention program, as gratitude and positive affect are viewed as protective psychological factors (Loseby et al., 2021). The positive effects of gratitude interventions on adolescents’ psychological well-being have been shown in previous research (Wood et al., 2010; Kwok et al., 2022). It will be beneficial to include exercises on positive affect and gratitude that are easy to incorporate into adolescents’ daily lives in an intervention. For example, participants can be asked to engage in activities that they enjoy, which will result in their experiencing positive emotions, thus facilitating the PERMA construct of engagement and positive emotions, and thereby acting as a facilitating condition to successful adjustment. This recommendation is in line with numerous studies indicating that positive psychological states are strongly associated with higher levels of participation in health behavior (Hingle et al., 2014; Huffman et al., 2015; Celano et al., 2020). Practical applications of the constructs, positive affect and gratitude, could be the inclusion of gratitude exercises and encouraging adolescents to engage in activities that elicit positive affect.

### Construct 4: support

Support was evident in theme 4 of the qualitative document analysis, “building a relationship with diabetes.” As support was identified as a crucial component of living positively with type 1 diabetes, it is recommended that caregivers should be involved during parts of the program. Research has shown that sharing experiences with people in similar situations decreases distress and improves mental health in adolescents and caregivers (Niela-Vilen et al., 2014; Ramchand et al., 2017).

In the qualitative document analysis, support from others, social media, and support groups was identified as being helpful in the process of adjustment. Therefore, support by participants of the program in the form of sharing their experiences should also be included in the intervention, as, in this way, they will motivate one another. Peer groups and online diabetes communities (e.g., blogs and social media) serve as a powerful source of support for people living with type 1 diabetes (Hilliard et al., 2015). In addition to assisting in the development of relationships with peers, social media generates a sense of belonging, reduces feelings of loneliness, increases the sharing of experiential knowledge and helps to reduce feelings of isolation (Wilson and Stock, 2021). It is recommended that within an intervention program, social support from various areas of the adolescents’ lives should be included, for example from social media, positive role models, friends, and family. Within the construct of support, the PERMA constructs of relationships and meaning are addressed and support is viewed as a facilitating condition to adjustment, in line with the UTAUT. Support is viewed as crucial in the process of adjustment to closed loop technology, as such, the inclusion of caregivers and peers are recommended in an intervention. Examples include, engaging in acts of kindness, spending quality time with people that they care about and caregivers given the task of emphasizing what they are proud of in terms of how the adolescent utilizes closed loop technology.

### Construct 5: trust

Within the second theme of the qualitative document analysis, “the process of positive adjustment to closed loop technology,” trust was an essential psychological construct. A lack of trust in diabetes technology is viewed as a barrier to successful adjustment (Tanenbaum and Commissariat, 2022). In the qualitative document analysis, trust in the closed loop system was facilitated when individuals adopted the view that the closed loop system was a helpful tool in managing the condition. Trust in the closed loop system was also facilitated by having access to visible glucose data. Within the qualitative document analysis, it was identified that trust is developed by creating a positive link between a self-care behavior and a visible change in glucose levels, which confirms a positive link between the behavior and the realization of the expected outcome. It is recommended that the concept of trust is included in interventions and that participants are encouraged to engage with their glucose data. It will be beneficial for participants to receive education on how to use glucose data to make informed diabetes management decisions. By addressing the construct of trust in an intervention, the PERMA aspect of engagement and the UTAUT construct of effort expectancy are included. A practical application could be empowering adolescents to use glucose data to obtain a better understanding of how closed-loop technology can benefit them and to increase their trust in the system.

## Implications and recommendations

Based on the findings from the qualitative document analysis, the focus of interventions that aim to assist with adolescents’ adjustment to closed loop technology should be on knowledge, responsibility, identity, positive affect, gratitude, support, and trust. Following this qualitative document analysis, an intervention will be developed by the current authors based on the psychological constructs identified within this article as the foundation of the intervention. The intervention will then be evaluated by a panel of experts, utilizing the Delphi method, as a part of the first author’s doctoral study. It is planned that the final intervention will also be discussed in a follow-up article.

## Limitations

The current study has shown that psychological factors play an important role in people’s adjustment to closed loop technology. Using a qualitative methodology was viewed as a strength of this study, as it enabled the authors to obtain an in-depth understanding of the experiences of those who are living with the condition. However, some limitations were identified; for example, all the participants in document 2 attended the same diabetes clinic. It is possible that the care received at another diabetes clinic can have a different impact on the process of adjustment. In addition, all the participants in this study controlled their diabetes well, as this was an inclusion criterion of the study in order to ensure homogeneity. It may be beneficial to explore the perspectives of people living with uncontrolled diabetes and to develop specific interventions for this group. It is possible that individuals with uncontrolled diabetes may have different lived experiences with diabetes technology. However, this study was aimed at obtaining an in-depth understanding of the experiences of adjustment to closed loop technology among adolescents with well-controlled type 1 diabetes. Document 2 also provided a richer account of the participants’ experience of adjustment as the results were supported by verbatim extracts in order for the reader to validate the results. Within document 1, the authors only had access to the published manuscript of the ongoing trial and did not have access to worksheets and a detailed layout of the intervention programme. Having access to the full programme could have provided a richer understanding of the programme, although the content of the programme is thoroughly explained in document 1. As document 1 is an ongoing trial, final results of the intervention effect is not yet available, thus the authors are not able to comment on the effectiveness of the intervention, however, document 1 was built on previous interventions, where the results were used to refine the intervention. The findings of the current study lay the foundation for possible interventions and other studies within the context of psychological factors and adjusting to closed loop technology.

## Conclusion

This qualitative document analysis aimed to identify essential constructs that need to be included in a positive psychology intervention to assist in adolescents’ adjustment to closed loop technology. The following key constructs should be addressed in the intervention: knowledge, responsibility, identity, positive affect, gratitude, support, and trust. Venkatesh and Davis’s (2000) UTAUT and Seligman’s (2011) PERMA model of well-being can be employed in the development of the intervention and used as the building blocks of the intervention, as these theories are relevant in the context of positive psychology and people’s adjustment to technology. Healthcare providers should be made aware of these constructs in order to assist adolescents in effectively adjusting to closed loop technology.

### Statement of contribution

Technological advancements in diabetes care, such as insulin pumps, continuous glucose sensors, and closed loop systems, offer hope and tools to improve outcomes in diabetes care (Gonder-Frederick et al., 2016). Despite these technological advances, glucose control has worsened in the adolescent population over the past decade, with adolescents showing low persistence in using these potentially enabling technologies (Bryden et al., 2001; Gonzalez et al., 2016). Although closed loop systems have many advantages, they do not work well for everyone living with diabetes, as the benefits of the technology depend largely on the individual’s engagement with the technology (Gonder-Frederick et al., 2016). A need exists to better understand and assist adolescents to benefit from closed loop technology. Positive psychology interventions in the context of type 1 diabetes may be beneficial, as such interventions would focus on the strengths of adolescents and their families to promote resilience. The key constructs identified in this qualitative document analysis are knowledge and education, responsibility, identity, positive affect, gratitude, support, and trust. The authors believe that the psychological constructs outlined in this document are a starting point in the development of interventions that assist adolescents with successful adjustment to closed loop technology.

## Data availability statement

The raw data supporting the conclusions of this article will be made available by the authors, without undue reservation.

## Ethics statement

The study was approved by the Health Research Ethics Committee of the North-West University, South Africa (NWU-00266-21-A1).

## Author contributions

SK: Conceptualization, Data curation, Formal analysis, Investigation, Methodology, Writing – original draft, Writing – review & editing. ED: Conceptualization, Formal analysis, Methodology, Supervision, Writing – review & editing. ER: Conceptualization, Methodology, Supervision, Writing – review & editing. DS: Writing – review & editing.

## Footnotes

^3^Kruger, S., Deacon, E., Van Rensburg, E., and Segal, D. G. (under review). Experiences of adjustment for adolescents living with well-controlled type 1 diabetes using closed loop technology. Health Psychol. Open.
